# Determining the influence of high glucose on exosomal lncRNAs, mRNAs, circRNAs and miRNAs derived from human renal tubular epithelial cells

**DOI:** 10.18632/aging.202656

**Published:** 2021-03-10

**Authors:** Sijie Zhou, Jiuyuan Fang, Mingyang Hu, Shaokang Pan, Dongwei Liu, Guolan Xing, Zhangsuo Liu

**Affiliations:** 1 Department of Nephrology, The First Affiliated Hospital of Zhengzhou University, Zhengzhou 450052, P. R. China; 2 Research Institute of Nephrology, Zhengzhou University, Zhengzhou 450052, P. R. China; 3 Research Center for Kidney Disease, Henan Province, Zhengzhou 450052, P. R. China; 4 Key Laboratory of Precision Diagnosis and Treatment for Chronic Kidney Disease in Henan Province, Zhengzhou 450052, P. R. China; 5 Core Unit of National Clinical Medical Research Center of Kidney Disease, Zhengzhou 450052, P. R. China; 6 Department of Pathology, The First Affiliated Hospital of Zhengzhou University, Zhengzhou 450052, P. R. China

**Keywords:** exosome, circRNA, lncRNA, miRNA, human renal tubular epithelial cells

## Abstract

Diabetic nephropathy is a lethal disease that can lead to chronic kidney disease and end-stage kidney disease. Exosomes, which are nanosized extracellular vesicles, are closely involved in intercellular communication. Most importantly, exosomes play critical roles in disease occurrence and development. However, the function of exosomes in diabetic nephropathy progression has not been fully elucidated. In the present study, we determined the expression profiles and differences of lncRNAs, mRNAs, circRNAs and miRNAs in exosomes derived from human renal tubular epithelial cells with or without high glucose (HG) treatment. A total of 169 lncRNAs, 885 mRNAs, 3 circRNAs and 152 miRNAs were differentially expressed in exosomes secreted by HG-challenged HK-2 cells (HG group) compared with controls (NC group). The functions of differentially expressed mRNAs, mRNAs colocalized or coexpressed with differentially expressed lncRNAs (DElncRNAs), potential target genes of miRNAs and source genes of circRNAs were investigated by Gene Ontology (GO) and Kyoto Encyclopedia of Genes and Genomes (KEGG) analysis.

According to these differentially expressed RNAs, we established an integrated circRNA-lncRNA-miRNA-mRNA regulatory network. In conclusion, our study suggested that exosomal lncRNAs, mRNAs, circRNAs and miRNAs participate in the progression of diabetic nephropathy and may be possible biomarkers and therapeutic targets in diabetic nephropathy.

## INTRODUCTION

Diabetic nephropathy (DN) is considered one of the most significant medical complications associated with diabetes. Approximately 1/4 of diabetes patients are inclined to develop DN [1]. The diagnosis of DN can only rely on the occurrence of at least microalbuminuria in a patient suffering from diabetes for over 5 years [2]. The current treatment of DN depended upon stabilizing the renin–angiotensin–aldosterone (RAAS) system by utilizing angiotensin converting enzyme inhibitors (ACEIs), angiotensin receptor blockers (ARBs), or aldosterone blockers (spironolactone or finerenone) [3]. However, the exacerbation of DN usually cannot be stopped because of the complicated molecular mechanisms involved in the initiation of renal injury in diabetes. Therefore, novel biomarkers that can facilitate earlier diagnosis and new therapeutic targets warrant further research.

Exosomes are specialized extracellular vesicles that consist of numerous types of DNA, RNA and proteins. Exosomes participate in the crosstalk between nearby and distant cells, and their diagnostic value and therapeutic potential are emerging [4, 5]. Additionally, the process of tumorigenesis may be slowed by inhibiting exocrine secretions [6]. Furthermore, long RNA species stabilized in exosomes, including mRNA, lncRNA and circRNA, can not only contribute to the progression of multiple diseases by regulating the behavior of recipient cells [7, 8] but can also be potential diagnostic biomarkers for human diseases, [9, 10] even for type 1 diabetes mellitus [11].

Tubular epithelial cells can also secrete exosomes. Studies have indicated that the delivery of CCL2 mRNA from tubular epithelial cell exosomes to macrophages results in severe kidney inflammation [12]. Most importantly, the use of exosomes derived from tubular epithelial cells may represent therapeutic strategies for kidney ischemia-reperfusion injury in rats [13]. In addition, recent studies have certified that exosomes play promising and critical roles in the diagnosis, progression and therapeutics of diabetes [7]. For example, exosomal miR-20b-5p can target AKT-interacting protein (AKTIP), thereby affecting AKT activity and reducing glycogen accumulation in primary human skeletal muscle, leading to insulin resistance [14]. Mesenchymal stem cell (MSC)-secreted exosome miR-146a functions as a protective factor against cognitive impairment caused by diabetes [15].

However, current studies exploring the role of exosomes secreted by tubular epithelial cells in diabetic nephropathy are still in their infancy, especially those involving lncRNAs and circRNAs. To explore the underlying molecular mechanism regulating exosomal noncoding RNAs in diabetic nephropathy, we investigated the expression profiles of exosomal lncRNAs, miRNAs, mRNAs, and circRNAs in high glucose-challenged HK-2 cells and normal controls. Our results may establish a foundation for future research investigating the use of exosomal noncoding RNAs in diagnosing and treating diabetic nephropathy.

## RESULTS

### Exosome enrichment and identification

**Figure 1:**
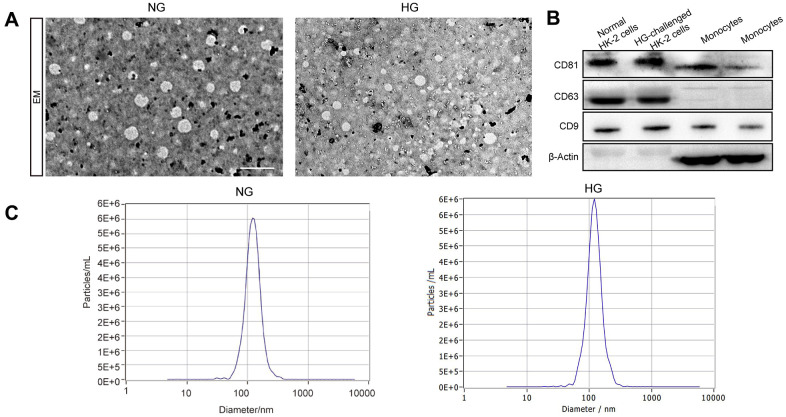
**The identification of exosomes.** Representative TEM image of exosomes derived from two groups (**A**). Western blot of the exosomal markers CD63, CD9, and CD81 and non-exosomal protein markers β-Actin in exosomes and monocytes (**B**). The size of the exosomes (nm) enriched from the culture supernatant of two groups was examined through NTA using a NanoSight NS300 instrument (NanoSight Ltd., Amesbury, UK) (**C**).

We first extracted exosomes from the culture supernatant of both the high-glucose-induced group and the control group. The characterization of exosomes, including their shape and size, was presented using TEM. The image indicates a round shape of exosomal vesicles and including size ranging from 47 to 154 nm (Figure 1A). We investigated the expression of the exosomal markers CD63, CD9, and CD81 in the exosomes isolated from these two groups and found them expressed in all of our samples (Figure 1B). Next, Exosome size was also identified by NTA, which indicated a mean size of 116.8nm in exosomes isolated from high glucose-induced HK-2 cells and 119.3nm in those isolated from normal HK-2 cells (Figure 1C). Taken together, our results indicated that the vesicles derived from high glucose-induced and normal HK-2 cells in line with the trend of exosome distribution.

### Differential expression analysis of exosomal lncRNAs, mRNAs, circRNAs and miRNAs

**Figure 2:**
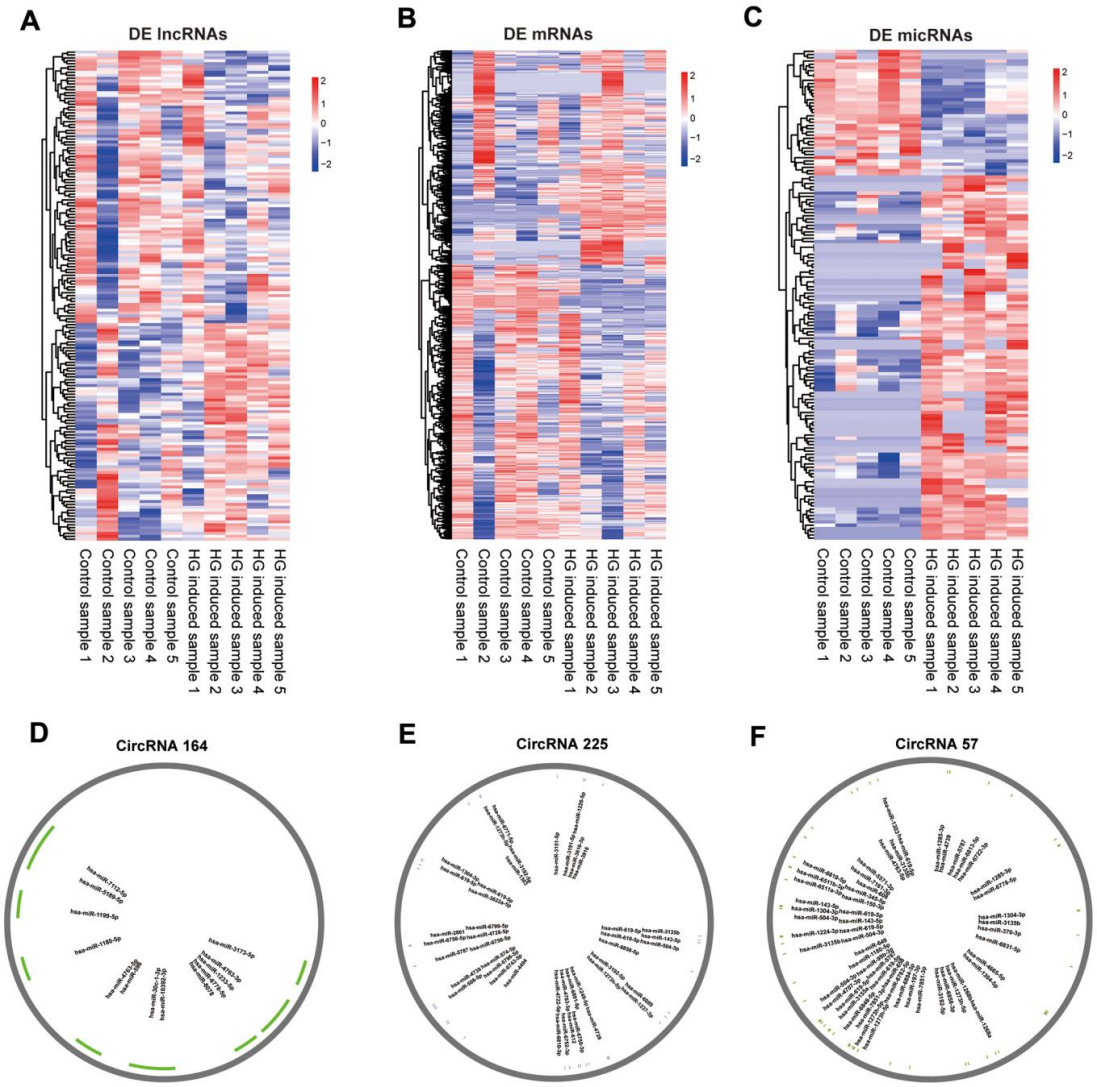
**Expression profiles of mRNAs, lncRNAs, miRNAs and pattern diagram of circRNAs /micRNA interaction prediction.** Hierarchical clustering of all differentially expressed exo-lncRNAs (**A**), exo-mRNAs (**B**), exo-miRNAs (**C**) and putative binding sites for miRNAs of circRNA_164 (**D**), circRNA_225 (**E**), circRNA_57 (**F**).

The whole-transcriptome sequencing data (lncRNA, circRNA, miRNA, mRNA) of five high-glucose induced samples and five control samples were obtained by hierarchical clustering. A total of 169 significantly expressed exo-lncRNAs were identified, including 93 upregulated lncRNAs and 76 downregulated lncRNAs (Figure 2A). The top five upregulated lncRNAs were RP11-178L8.9, CTD-2530H12.2, RP11-503N18.4, RP11-20B24.7 and RP11-256I23.1, and the top five downregulated lncRNAs were RP11-517A5.7, RN7SL870P, CTD-2298J14.2, ANKRD10-IT1 and AP000442.1. In addition, a total of 885 exo-mRNAs were differentially expressed (Figure 2B). Among these exo-mRNAs, 403 were upregulated and 482 were downregulated in the HG groups compared with the control groups. Meanwhile, the top five upregulated mRNAs included CASP7, ZNF766, AL603965.1, ELK3 and MRPL36, and downregulated mRNAs included FASLG, MRPL42, AL137002.1, CLTC and MT-ND6. In the same way, 152 exo-microRNAs were identified as being differential expressed (Figure 2C). Of these exo-microRNAs, 113 were identified as upregulated, and 39 were identified as downregulated. The top five upregulated microRNAs were hsa-miR-6724-5p, hsa-miR-6716-3p, hsa-miR-2355-3p, hsa-miR-135b-3p and hsa-miR-3180. The top 5 downregulated microRNAs were hsa-miR-5008-3p, hsa-miR-6785-5p, hsa-miR-3654, hsa-miR-335-3p and hsa-miR-3074-3p. Finally, only 3 circRNAs were specifically dysregulated, and they were all downregulated in the HG groups, including circRNA_164, circRNA_225 and circRNA_57. Information on these identified circRNAs is presented in Table 1. To further predict the function of differentially expressed exo-circRNAs, we established the circRNA/microRNA interaction based on the Circular RNA Interactome database (https://circinteractome.nia.nih.gov/).

**Table 1:** The information and annotation of identified DE exo-circRNAs.

**CircRNA ID**	**Chr**	**Known_circRNA_ID**	**Full length**	**Gene**	**Transcript**	**Feature**
**circRNA_164**	chr12	hsa_circ_0028717; hsa_circ_0000448	750	GCN1	ENST00000548132.1_1	whole_trans
**circRNA_225**	chr21	hsa_circ_0061273; hsa_circ_0141673; hsa_circ_0004771	29231	NRIP1	ENST00000318948.6_1	exonic
**circRNA_57**	chr3	hsa_circ_0068682; hsa_circ_0008583	28619	DLG1	ENST00000346964.6_2	exonic

### Functional enrichment analysis

**Figure 3:**
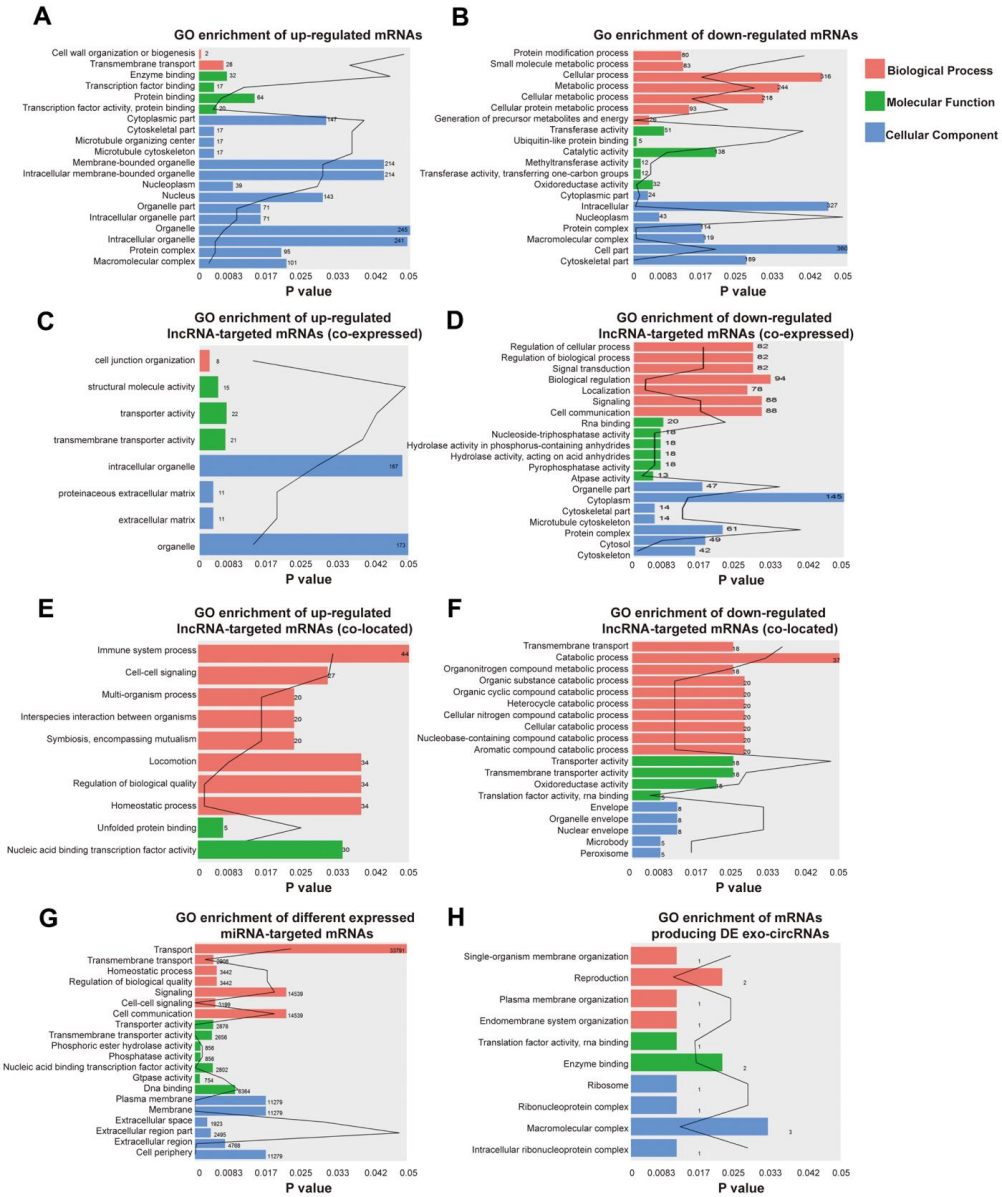
**Identification the biological role of DE exo-mRNAs, lncRNAs, circRNAs and miRNAs by GO analysis.** Go terms of up- (**A**) and down- (**B**) regulated exo-mRNAs, up- (**C**) and down- (**D**) regulated exo-lncRNA co-expressed mRNAs, up- (**E**) and down- (**F**) regulated exo-lncRNA co-located mRNAs, up- and down-regulated mRNAs targeted by exo-miRNAs (**G**) and derived from DE exo-circRNAs (**H**). We only presented top 20 terms with p-value under 0.05.

GO enrichment analyses were conducted to explore the potential function of differentially expressed exo-lncRNAs, mRNAs, circRNAs and miRNAs. GO classification showed that 2 biological processes (BPs), such as transmembrane transport, 4 molecular functions (MFs), such as transcription factor activity-protein binding, and 17 cellular components (CCs), such as macromolecular complex, were the major functions of these upregulated exo-mRNAs (Figure 3A). Twenty biological processes (BPs), such as cellular metabolic process, 6 molecular functions (MFs), such as methyltransferase activity, and 27 cellular components (CCs), such as cell part, were the major functions of these downregulated exo-mRNAs (Figure 3B). In addition, under the hypothesis that exo-lncRNA function could be associated with the known function of their targeted mRNAs, including colocated and coexpressed mRNAs, lncRNA-targeted mRNAs were further mapped with GO terms. The results show that GO items of upregulated exo-lncRNA-targeted mRNAs (coexpressed) were involved in 1 biological process (BP) called cell junction organization, 3 molecular functions (MFs), such as transporter activity, and 4 cellular components (CCs), such as extracellular matrix (Figure 3C). GO items of downregulated exo-lncRNA-targeted mRNAs (coexpressed) were involved in 30 biological processes (BPs), such as signal transduction and regulation of biological process, 6 molecular functions (MFs), such as pyrophosphatase activity and RNA binding, and 13 cellular components (CCs), such as protein complex (Figure 3D).

Moreover, GO items of upregulated exo-lncRNA-targeted mRNAs (colocated) were involved in 8 biological processes (BPs), such as homeostatic processes, and 2 molecular functions (MFs), such as nucleic acid binding transcription factor activity (Figure 3E). GO items of downregulated exo-lncRNA-targeted mRNAs (colocated) were involved in 10 biological processes (BPs), such as cellular catabolic process and transmembrane transport, 4 molecular functions (MFs), such as translation factor activity and RNA binding, and 5 cellular components (CCs), such as nuclear envelope (Figure 3F).

Functional enrichment analysis based on the differentially expressed exo-miRNAs indicated that the mRNAs targeted by upregulated and downregulated miRNAs were related to 18 biological processes (BPs), such as cell-cell signaling and homeostatic process, 7 molecular functions (MFs), such as DNA binding, and 6 cellular components (CCs), such as the plasma membrane (Figure 3G).

According to the relationship between circRNAs and source genes, GO analyses were also performed to investigate the potential function of differentially expressed exo-circRNAs. The mRNAs producing differentially expressed exo-circRNAs were related to 4 BPs, such as single-organism membrane organization, 2 MFs, such as enzyme binding, and 4 CCs, such as macromolecular complexes (Figure 3H).

**Figure 4:**
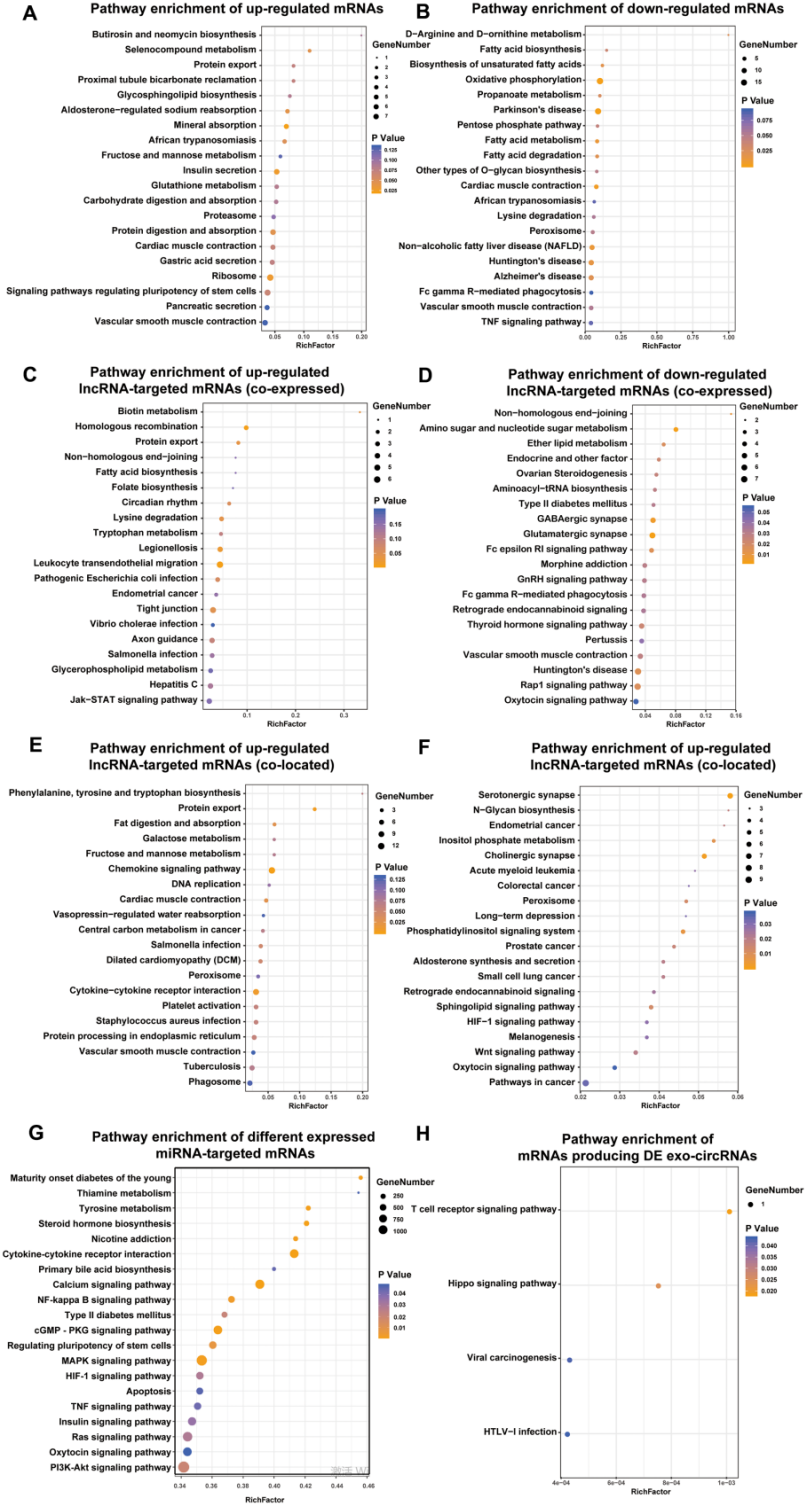
**Identification the biological role of DE exo-mRNAs, lncRNAs, circRNAs and miRNAs by KEGG analysis.** KEGG pathways of up- (**A**) and down- (**B**) regulated exo-mRNAs, up- (**C**) and down- (**D**) regulated exo-lncRNA co-expressed mRNAs, up- (**E**) and down- (**F**) regulated exo-lncRNA co-located mRNAs, mRNAs targeted by DE exo-miRNAs (**G**) and derived from DE exo-circRNAs (**H**). We only presented top 20 terms.

To further explore the potential biological role of the identified exo-lncRNAs, mRNAs, circRNAs and miRNAs, we utilized KEGG to provide functional annotations of genes based on their associated biochemical pathways. Enrichment analysis for significantly differentially expressed mRNAs showed that upregulated mRNAs were related to 7 pathways, including insulin secretion, protein digestion and absorption (Figure 4A), and downregulated mRNAs were associated with 15 pathways, including fatty acid metabolism and fatty acid degradation (Figure 4B).

With respect to differentially expressed lncRNA-targeted mRNAs (colocated and coexpressed mRNAs), up- and downregulated lncRNA-targeted mRNAs (coexpressed) were related to such pathways as biotin metabolism, type II diabetes mellitus, amino sugar and nucleotide sugar metabolism, and the GnRH signaling pathway (Figure 4C–4D). Up- and downregulated lncRNA-targeted mRNAs (colocated) were related to such pathways as the chemokine signaling pathway, fat digestion and absorption, N-glycan biosynthesis and HIF-1 signaling pathway. In addition, mRNAs targeted by the up- and downregulated exo-miRNAs were associated with such pathways as maturity onset diabetes of the young, type II diabetes mellitus, and insulin signaling pathway (Figure 4G). Furthermore, mRNAs producing DE exo-circRNAs were associated with such pathways as the Hippo signaling pathway and T cell receptor signaling pathway (Figure 4H).

Most importantly, the defined DE exo-RNAs were frequently enriched in protein export pathway, which can provide strong evidence for the imperative role of exosomes in transporting proteins. These proteins may be secreted by exosomes and participate in the progression of DN occurrence and development.

### CeRNA network of DE exo-mRNAs, lncRNAs, circRNAs and miRNAs

**Figure 5:**
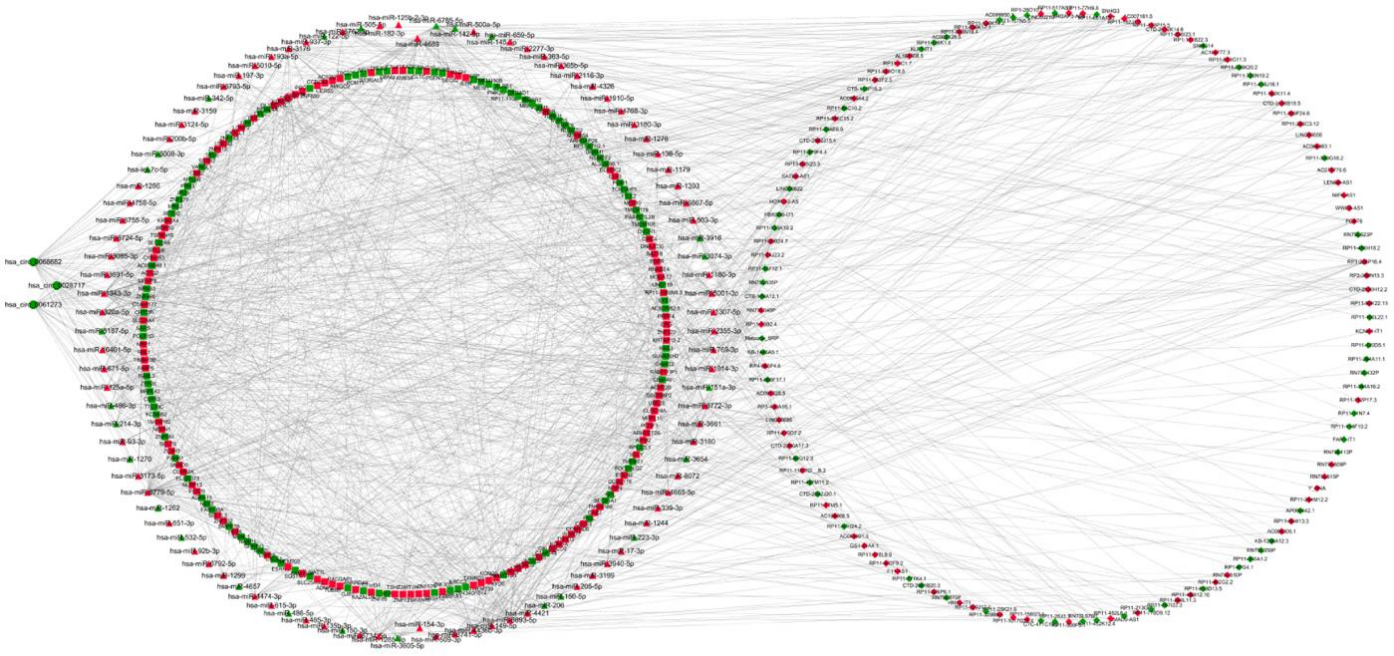
**LncRNAs-circRNAs-miRNAs-mRNAs regulation network.** DElncRNAs are depicted by diamonds, DEmRNAs are indicated by the square, DEcircRNAs are represented by circle and DE miRNAs are presented by triangle. The red represents up-regulated genes whereas green represents down-regulated genes.

To investigate the interaction between the exo-lncRNAs, circRNAs and miRNAs and mRNAs, the circRNA-lncRNA-miRNA-mRNA network was constructed according to the ceRNA hypothesis by integrating expression profile data and their regulatory relationships. We first utilized miRanda and TargetScan software to predict miRNA binding sites of the DE exo-circRNA and exo-lncRNAs. Then, we extracted differentially expressed miRNAs from the intersection of these predicted miRNAs targeted by DE exo-circRNAs and exo-lncRNAs. Following this step, we forecasted DE exo-mRNAs that were potential targets of these DE miRNAs. Finally, a network of DE exo-circRNA-lncRNAs-miRNAs-mRNAs, including a total of 3 circRNAs, 137 lncRNAs, 102 miRNAs and 207 mRNAs, was visualized by Cytoscape (Figure 5).

**Figure 6:**
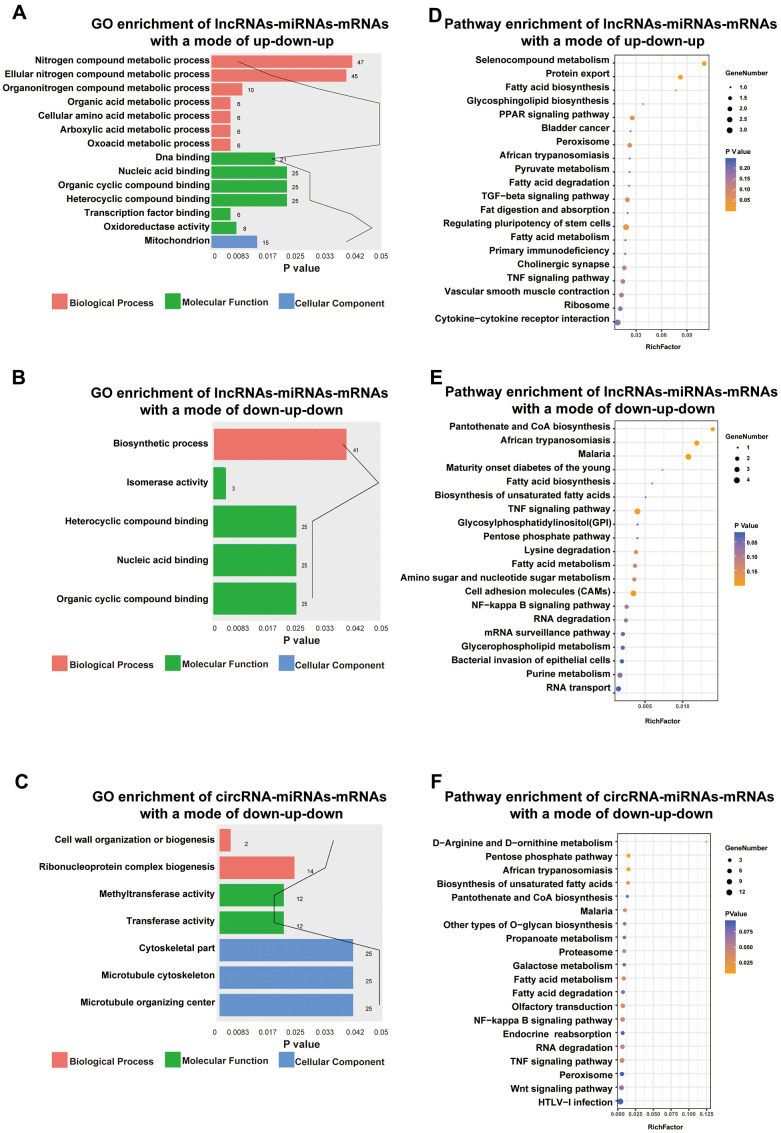
**Functional enrichment analysis towards this ceRNA network.** Go annotation of lncRNAs-miRNAs-mRNAs with a mode of up-down-up (**A**) or down-up-down (**B**) and circRNA-miRNAs-mRNAs with a mode of down-up-down (**C**). KEGG pathway enrichment analysis of lncRNAs-miRNAs-mRNAs with a mode of up-down-up (**D**) or down-up-down (**E**) and circRNA-miRNAs-mRNAs with a mode of down-up-down (**F**) with top 20.

Functional enrichment analysis indicated that DE lncRNAs/circRNAs-miRNAs-mRNAs with an up-down-up expression pattern were related to BPs, such as oxoacid metabolic process, MFs, such as DNA binding, and CCs, such as the mitochondria (Figure 6A). Additionally, DE lncRNAs-miRNAs-mRNAs with a mode of down-up-down were related to BPs, such as biosynthetic processes, and MFs, such as nucleic acid binding (Figure 6B). DE circRNAs-miRNAs-mRNAs with a mode of down-up-down were associated with BPs, such as cell wall organization or biogenesis, MFs, such as methyltransferase activity, and CCs, such as cytoskeletal part (Figure 6C).

Moreover, KEGG pathway enrichment analysis for lncRNAs/circRNAs-miRNAs-mRNAs with an up-down-up expression pattern indicated that such pathways as the PPAR signaling pathway and protein export (Figure 6D) were enriched. However, lncRNAs-miRNAs-mRNAs with a mode of down-up-down were enriched in such pathways as the TNF signaling pathway and cell adhesion molecules (CAMs) (Figure 6E). Moreover, circRNAs-miRNAs-mRNAs with a mode of down-up-down were enriched in such pathways as the NF-kappa B signaling pathway and fatty acid metabolism (Figure 6F).

Previous study had certificated some molecules could aggravate renal injury in diabetic nephropathy through the TNF-α pathway [16, 17]. In our study, we found that mRNAs in the sub-ceRNA network all enriched in TNF-α signaling pathway, which indicated the exosomal RNAs derived by high glucose-challenged HK-2 cells may contribute to DN progression via activating TNF-α pathway.

## DISCUSSION

Diabetic nephropathy (DN) is currently the most serious complication associated with diabetes mellitus. The standard therapeutic intervention for diabetic kidney disease is the application of an angiotensin-converting enzyme inhibitor or angiotensin receptor blocker—a strategy that has been emerging for over two decades [18]. Unfortunately, the occurrence of end-stage renal disease elevates the risk of kidney failure, and mortality is observed to increase with diabetes by 12-fold [19].

With the help of large-scale genome-sequencing technologies, recent advances had produced large numbers of molecular biomarkers in DN [20, 21]. Exosomes are common membrane-bound nanovesicles that consist of various biomolecules. Recent studies have certified the critical role of exosomes in DN. For instance, exosomes secreted by high glucose-treated glomerular endothelial cells contributed to promoting epithelial mesenchymal transition (EMT) and fibrosis of glomerular mesangial cells and podocytes in DN [22, 23]. The expression of urinary exosomal WT1 was increased in type 2 DN patients compared to healthy patients, which may facilitate the diagnosis of type 2 DN [24]. Exosomes derived from tubular epithelial cells can also transport functional cargos to receptor cells and play biological roles in receptor cells [25]. However, the exact molecular biological roles of such cell-to-cell communication in DN remain largely unknown, and the lack of effective diagnostic biomarkers for DN remains a major problem.

In the present study, we obtained a total of 69 DE exo-lncRNAs, 885 DE exo-mRNAs, 3 DE exo-circRNAs and 152 DE exo-miRNAs in HG-challenged HK-2 cells (HG group) compared with controls (NC group). Among these differentially expressed mRNAs in exosomes, CAMK2B has been found to be overexpressed in the liver of STZ-induced male diabetic mice and is involved in the pathogenesis of diabetes [26]. Shibasaki et al. indicated that RAPGEF4(Epac2)/Rap1 signaling participated in augmentation of the first phase of insulin secretion [27]. The application of Epac2 agonists might be helpful for the treatment of type 2 diabetes with impaired insulin release from pancreatic β-cells [28]. Proteomic analysis of exosomes derived from primary human proximal tubular epithelial cells under normal and inflammation culture conditions showed that TF was upregulated between inflammation versus normal, unbiased pathway analyses TF is correlated with “Renal Impairment” [25]. Our study indicated that TF is upregulated in the HG groups, which verified its potential function in renal injury. In addition, miR-486-5P was downregulated in the HG groups. Viñas et al. [29] proposed that exosome miR-486-5P secreted by human endothelial colony–forming cells (ECFCs) is a key paracrine factor protecting against ischemic cell death and loss of kidney function, which suggests a new therapy method by exosomes. Moreover, among these three DE exo-circRNAs, hsa_circ_0004771 was involved in the progression of multiple cancers and could be used as a diagnostic biomarker for colorectal cancer [30–32]. However, there are few investigations about the function of exosomal lncRNAs and circRNAs in DN, and the specific mechanism of these three circRNAs in DN has not been determined.

GO enrichment analysis showed that DE exo-mRNAs were associated with such terms as transmembrane transport, cellular metabolic process and transferase activity. DE exo-miRNAs were related to terms including cell-cell signaling, cell communication and homeostatic process. DE exo-lncRNAs (colocated and coexpressed) were associated with such terms as extracellular matrix, immune system process, catabolic process and signal transduction. DE exo-circRNAs were related to terms including translation factor activity and RNA binding. Among these GO terms, the extracellular matrix is a fibrosis-related biological process that plays a critical role in renal injury caused by DN [33]. Such GO terms as cell-cell signaling and cell communication supported the imperative function of exosomes in cell interactions.

KEGG pathway enrichment analysis showed that differentially expressed exo-RNAs were associated with such pathways as insulin secretion and fatty acid metabolism. DE exo-miRNAs were related to pathways including cytokine-cytokine receptor interaction, type II diabetes mellitus, hedgehog signaling pathway and maturity onset diabetes of the young. DE exo-lncRNAs (colocated and coexpressed) were associated with such pathways as the chemokine signaling pathway, HIF-1 signaling pathway, and type II diabetes mellitus. DE exo-circRNAs were related to pathways including the Hippo signaling pathway. Among these enriched pathways, cytokine-cytokine receptor interactions and HIF-1 signaling pathways have been reported to play an important role in the progression of DN [33, 34]. In addition, the activation of the Hedgehog signaling pathway contributes to interstitial fibroblasts after kidney injuries [35]. The ceRNA hypothesis proposes that circRNA/lncRNAs can regulate gene expression by interacting with miRNA at miRNA-binding sites (MREs) [36]. To the best of our knowledge, our study is the first to establish an exosomal lncRNA/circRNA-miRNA-mRNA ceRNA network of HG-induced HK2 cells based on our RNA-seq data, and we found 3 circRNAs, 137 lncRNAs (e.g., PCAT6, LINC00210, LINC00622), 102 miRNAs (e.g., hsa-miR-182-3p, hsa-miR-1910-5p, hsa-miR-1914-3p, hsa-miR-193a-5p) and 207 mRNAs (e.g., BAZ1B, CFL2, ERG). Furthermore, our research has several limitations. First, the sample (5 control samples and 5 HG-induced samples) consisted of cell samples instead of human subjects, raising the issue of repeatability in human tissues. Second, although we screened differentially expressed lncRNAs, circRNAs, miRNAs, and mRNAs in exosomes, their specific functions have not been investigated or verified.

In conclusion, our study indicated significant differences in the expression of exosomal lncRNAs, mRNAs, circRNAs, and miRNAs between high glucose-challenged HK-2 cells and normal controls, and the functions of these RNAs were also determined according to our RNA-seq analysis. These differentially expressed RNAs may be employed as biomarkers and therapeutic targets in diabetic nephropathy.

## MATERIALS AND METHODS

### Cell culture

The human proximal tubular epithelial cell line HK2 was cultured in DMEM plus F12 (1:1) medium (Sigma-Aldrich, St. Louis, MO, USA) supplemented with 10% FBS (Gibco, USA) and 1% penicillin (100 U/ml)-streptomycin (100 μg/ml) (Gibco, USA) at 37° C in a humidified atmosphere of 5% CO_2_. For glucose stimulation experiments, the cells were cultured in high glucose (HG) medium containing 30 mmol/l D glucose for 48 h at 37° C. For the control group, HK2 cells were cultured in ordinary medium. Fresh medium was supplied every 3 days. Five duplicates of each group were prepared. Culture supernatants of both the high glucose-induced group and the control group were collected for further exosome isolation.

### Exosome extraction

Cell supernatant from each sample was collected for exosome enrichment using the ultracentrifugation method. The cell supernatant was centrifuged at 300 g for 10 min to eliminate cell components. The supernatant was further centrifuged at 2,000 g for 10 min to eliminate dead cells, and then we eliminated debris and shed microvesicles by centrifuging at 10000 g for 30 min and at 100000 g for 70 min sequentially. Then, the supernatant was further centrifuged at 100000 g for 70 min to eliminate contaminating proteins. Finally, the pellet was resuspended in PBS and stored at -80° C for further use.

### Transmission electron microscopy (TEM)

The exosome suspension was diluted to 0.5 mg/ml suspension with 1X PBS at 4° C. The suspension was then added onto the carbon-coated nickel mesh placed on filter paper. After 30 min of fixing under infrared light, the samples were stained with one drop of phosphotungstic acid (1% aqueous solution) and dried again for 5 min. Finally, the morphology of exosomes was visualized by a transmission electron microscope (HT7700, Hitachi, Tokyo, Japan).

### Nanoparticle tracking analysis (NTA)

Exosomes were diluted and analyzed with a NanoSight NS300 instrument according to the manufacturer's instructions. NTA 3.2 software records the particle trajectory and computes the concentration and diameter distribution of all samples. The exosome concentration of the original solution was calculated based on the dilution.

### Western blot analysis

Exosomes were further identified by western blotting. We selected blood monocytes as non-exosome controls. First, ice-cold 1X RIPA buffer (Thermo Scientific, Pittsburgh, PA) was utilized to lyse exosome proteins, and the protein concentration of exosomes was quantified using a BCA protein assay kit (Beyotime, China). Protein samples were separated using 8% SDS-PAGE and transferred to PVDF membranes. The PVDF membranes were blocked with 5% BSA for 1 h, washed 3 times (10 min each time) with 1X TBST and probed overnight with primary antibodies against the most common exosomal proteins CD63 (Abcam, USA), CD9 (Abcam, USA), CD81 (Cell Signaling Technology, USA) and non-exosomal protein markers β-Actin (Abcam, USA) at 4° C. Membranes were then washed and incubated with a secondary antibody at room temperature for 1 h. Finally, the membrane was covered with an enhanced chemoluminescence detection kit (GE Healthcare, Piscataway, NJ) to display protein bands.

### Exosomal RNA isolation, RNA library preparation and sequencing

Total exosomal RNA from each sample was extracted by TRIzol reagent (Invitrogen, USA) according to the manufacturer’s instructions. The quality and purity of the extracted RNAs were measured using an Agilent 2100 Bioanalyzer (Agilent Technologies, USA). The sequencing library was performed using NEXTflex®Small RNA-Seq Kit v3 (Bio Scientific Corporation, NOVA-5132-05) or Ovation Human FFPE RNA-Seq Library System (NUGEN, 0340-32), and we used a sample input of 20 ng of each total RNA. Finally, we profiled the expression of the sequencing library using HiSeq 2500 (Illumina, Inc., San Diego, CA, USA). Cutadapt [37] software was utilized to remove low-quality reads and acquire high-quality reads. The comparison software Tophat (http://ccb.jhu.edu/software/tophat/index.shtml) was used to map clean reads to a reference genome annotated with a gene location, and Bowtie2 (http://ccb.jhu.edu/software/tophat/index.shtml) was applied to build the index of the reference genome. CircRNA was identified by find_circ [38] and CIRCexplorer [39] through corresponding strict filtering. We used StringTie software to assemble the transcripts and utilized Perl scripts to screen known lncRNAs. Quantitative analysis of lncRNAs and mRNAs was performed using the ballgown R package.

### Differential expression analysis

Differential exosomal mRNA, lncRNA, miRNA and circRNA expression analysis of the two groups was conducted by DEGseq [40]. Fold change (FC) was regarded as an indicator of differential expression between the HG-induced group and the control group. T-tests were utilized to evaluate the statistical significance of differences. P-values < 0.05 were considered to demonstrate differential expression.

### Gene ontology (GO) and kyoto encyclopedia of genes and genomes (KEGG) enrichment analysis

Gene Ontology (GO) enrichment analysis was conducted according to the target gene candidates of differentially expressed miRNAs, the source genes of differential circRNAs and colocated or coexpressed genes with differential lncRNAs. In addition, we applied the NCBI DAVID server (http://david.abcc.ncifcrf.gov) with default settings [41] to test the statistical enrichment of the target gene candidates in KEGG pathways.

### Target gene prediction and competing endogenous RNA (ceRNA) network construction

Detailed circRNA information was downloaded from the circBase database (http://www.circbase.org/). The target mRNAs of miRNAs were theoretically evaluated by TargetScan (http://www.targetscan.org/vert_71/), RNAhybrid (http://bibiserv.techfak.uni-bielefeld.de/rnahybrid) and miRanda (https://regendbase.org/tools/miranda).
